# Revisiting a Case of Parathyroid Adenoma With Bilateral Staghorn Calculus

**DOI:** 10.7759/cureus.8251

**Published:** 2020-05-23

**Authors:** Manish Gupta, Habibulla Khan, Vijay S Nijhawan, Saurabh Gaba, Monica Gupta

**Affiliations:** 1 Otorhinolaryngology, Maharishi Markandeshwar Institute of Medical Sciences and Research, Ambala, IND; 2 Otorhinolaryngology, Maharishi Markandeshwar Institute of Medical Science and Research, Ambala, IND; 3 Pathology, Maharishi Markandeshwar Institute of Medical Science and Research, Ambala, IND; 4 General Medicine, Government Medical College and Hospital, Chandigarh, IND

**Keywords:** parathyroid gland adenoma, staghorn calculus, primary hyperparathyroidism, minimally invasive parathyroidectomy

## Abstract

Parathyroid gland adenoma is responsible for approximately 80%-85% cases of primary hyperparathyroidism. Although not much is diagnostic on clinical examination, the blood investigations of the patients reveal raised serum calcium and serum parathyroid hormone levels. We present a case of chronic kidney disease with bilateral staghorn calculi and a right parathyroid adenoma localized on ultrasonography. Parathyroid adenoma was surgically excised by minimally invasive parathyroidectomy.

## Introduction

Parathyroid proliferative disease (PPD) is a collective term encompassing all the histological variants which causes hyperparathyroidism, i.e., parathyroid hyperplasia, parathyroid adenoma, and parathyroid carcinoma [1]. Hyperparathyroidism has three forms: primary, secondary, and tertiary. Patients with primary hyperparathyroidism (PHPT) have elevated serum calcium and serum parathyroid hormone (PTH) levels. Parathyroid adenoma is responsible for 85% cases, followed by 14% of parathyroid hyperplasia and rarely (1%) of parathyroid carcinoma [1]. Parathyroid hyperplasia is usually seen in patients with chronic renal disease, resulting in secondary hyperparathyroidism. The independent, uncontrolled secretion of parathormone in cases of long-standing renal disorders is responsible for tertiary hyperparathyroidism.

PHPT results in abnormal calcium homeostasis due to the inappropriate or unregulated overproduction of PTH. These high levels promote increased renal calcium resorption and phosphate excretion, greater 1,25(OH)2D synthesis, and increased bone resorption. PHPT is often referred to as the ‘stone and bone’ disease. PHPT has multiple renal manifestations, which may include hypercalciuria, nephrolithiasis, nephrocalcinosis, chronic kidney disease, and renal tubular dysfunction. Due to the routine biochemical panel-based screening, PHPT is typically incidentally diagnosed. However, India still continues to see the symptomatic variant of PHPT [2]. Despite advances in the medical management, parathyroidectomy is the only curative approach to this disease. Individuals who refuse surgery or where it is not possible or desirable, skeletal protection and lowering of serum calcium is indicated [3].

## Case presentation

A 35-year-old female was referred to ear, nose and throat (ENT) outpatient department from urology department where she was being evaluated for bilateral renal calculus. The patient had complaints of bilateral flank pain, hematuria, and generalized weakness. There was no history of neck swelling, bony pains, repeated fractures, visual disturbances, or any neuropsychiatry illness. There was no history of weight loss, anorexia, diarrhea or constipation, amenorrhea, and galactorrhea. There was no family history of similar disease. On examination, the patient was thin built with mild pallor. ENT examination was within normal limits. Neck examination revealed no abnormal swelling.

**Figure 1:**
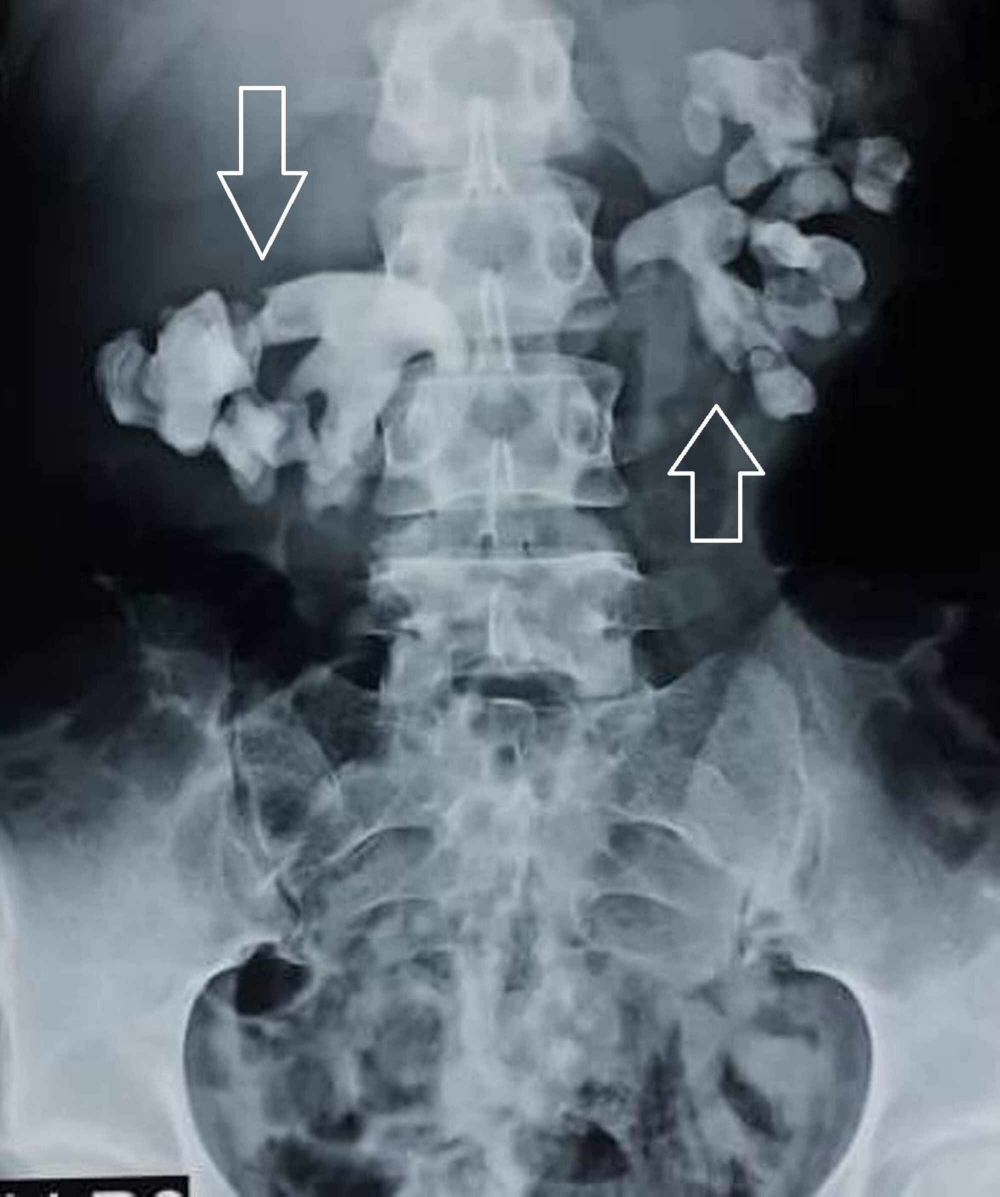
X-ray kidney ureter bladder showing bilateral staghorn renal calculi (white arrows).

Laboratory data revealed a low hemoglobin of 8.0 gm/dl, normocytic normochromic with normal white blood cell and platelet counts, elevated blood urea nitrogen and serum creatinine (46.80 and 2.73 mg/dl, respectively), and raised serum calcium of 12.4 mg/dl (reference range: 8.5-10.5 mg/dl) with corresponding serum albumin of 4.1 gm/dl, raised serum phosphorous of 4.65 mg/dl (reference range: 2.5-4.5 mg/dl), and elevated intact parathormone of 305 pg/ml (reference range: 14-72 pg/ml). The 25-hydroxyvitamin D level was 35 ng/ml. The rest of blood investigations were within normal ranges. Abdominal kidney ureter bladder (KUB) plain film radiograph showed bilateral radiopaque, branched stone, filling the renal pelvis and calyces (staghorn calculus; Figure 1).

**Figure 2:**
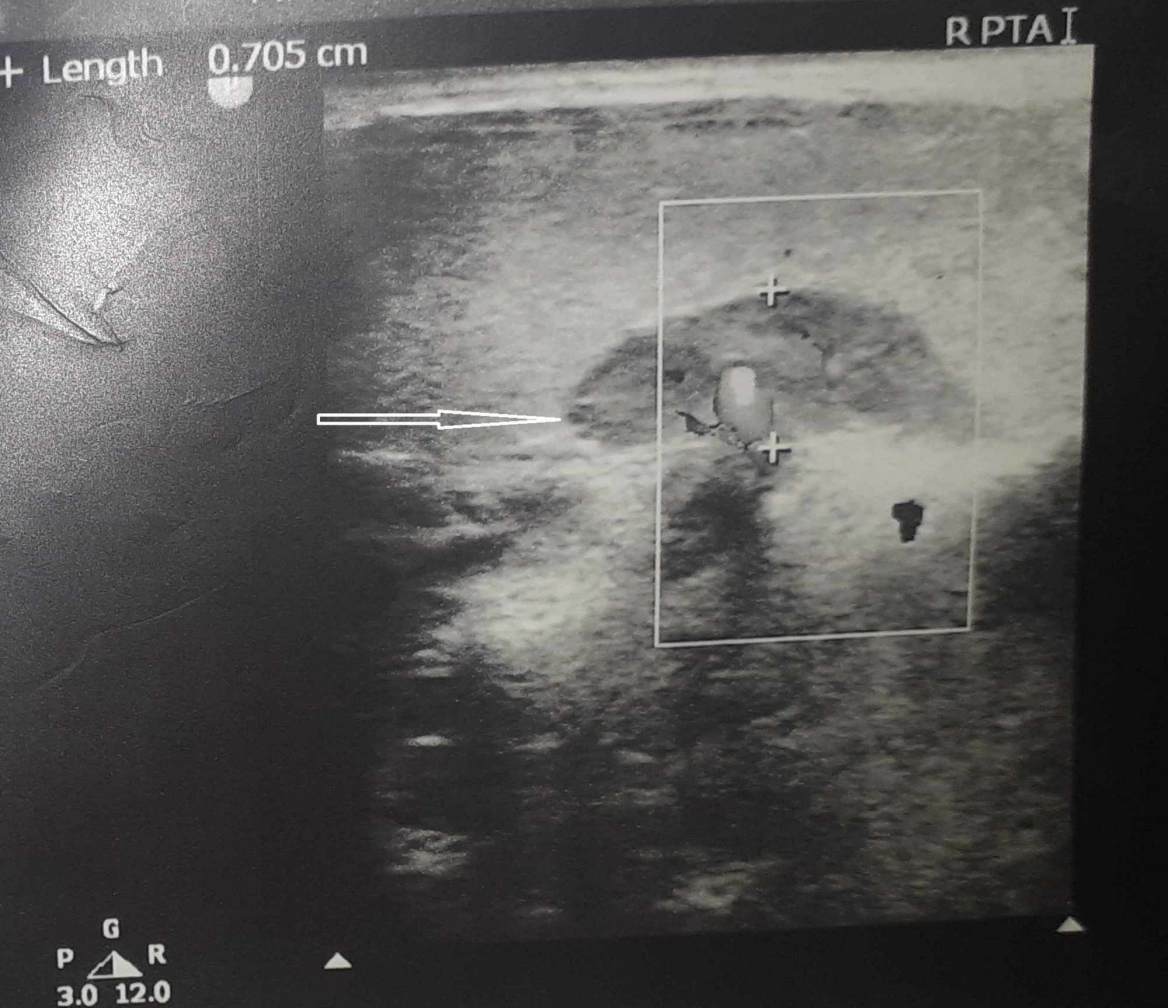
High-resolution ultrasound image showing a well-defined hypoechoic mass of 14x7 mm in diameter (white arrow).

High-resolution ultrasonography showed a well-defined hypoechoic mass of 14x7 mm in diameter behind the right lobe of thyroid gland (Figure 2).

**Figure 3:**
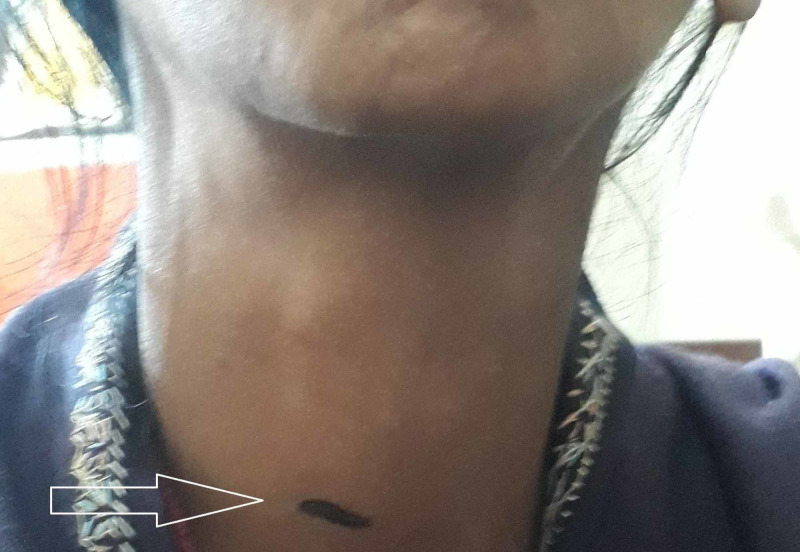
Preoperative skin marking of right superior parathyroid gland, as confirmed by ultrasonography (white arrow).

With ultrasound preoperative localization, skin marking of incision was made and the patient was planned for minimally invasive parathyroidectomy (MIP) (Figure 3).

**Figure 4:**
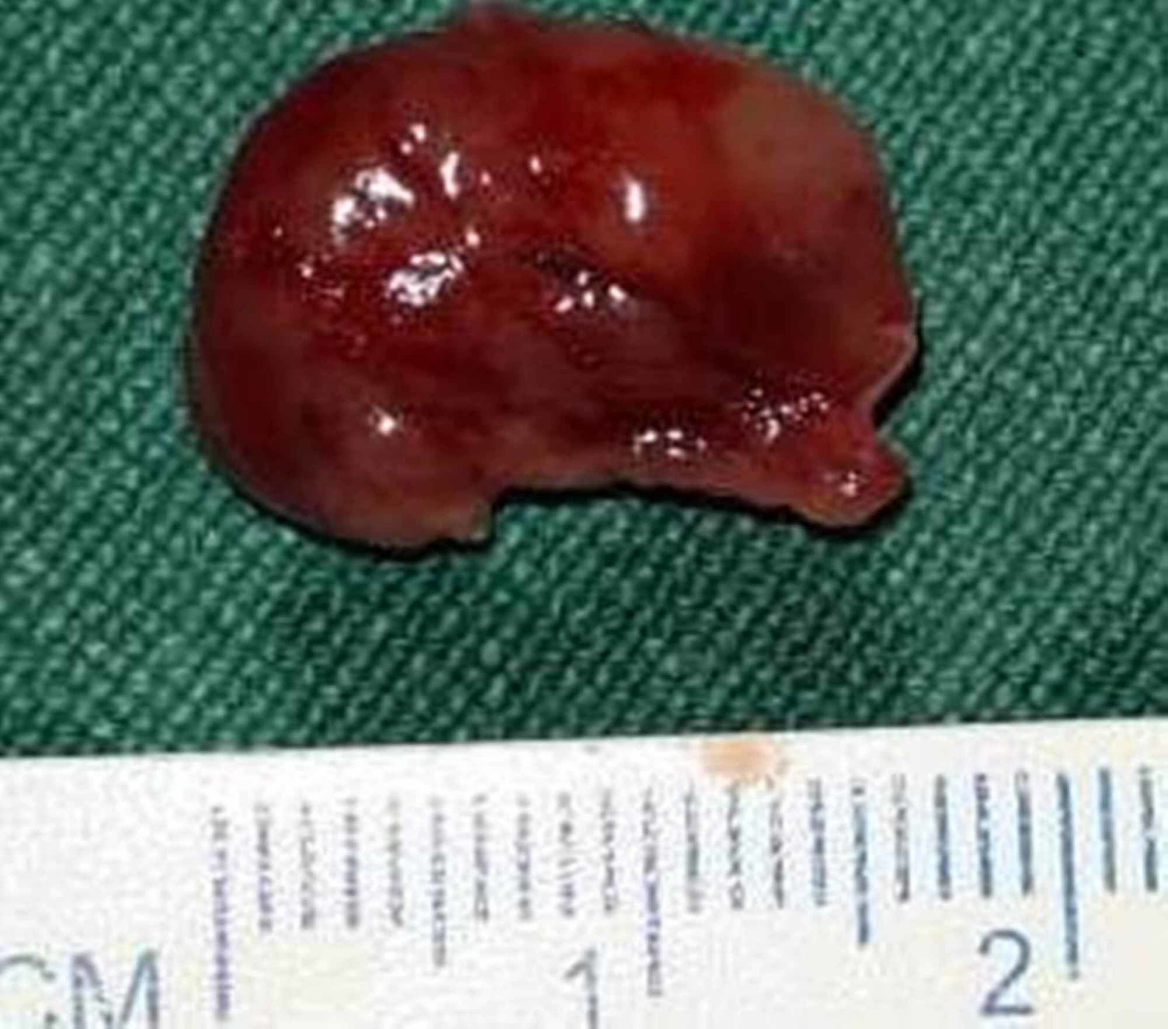
Gross picture of excised parathyroid mass.

Low blood hemoglobin was corrected with stored packed red blood cells transfusion prior to surgery. Using a small neck crease incision, the middle portion of right lobe thyroid gland was approached. The tumor was found originating from the superior right parathyroid gland. The tumor was resected completely (Figure 4), and an intraoperative PTH monitoring was done after 10 minutes post-excision which showed a PTH level of 103 pg/ml (Miami criteria: >50% PTH drop compared to pre-incision measurement) [4].

**Figure 5:**
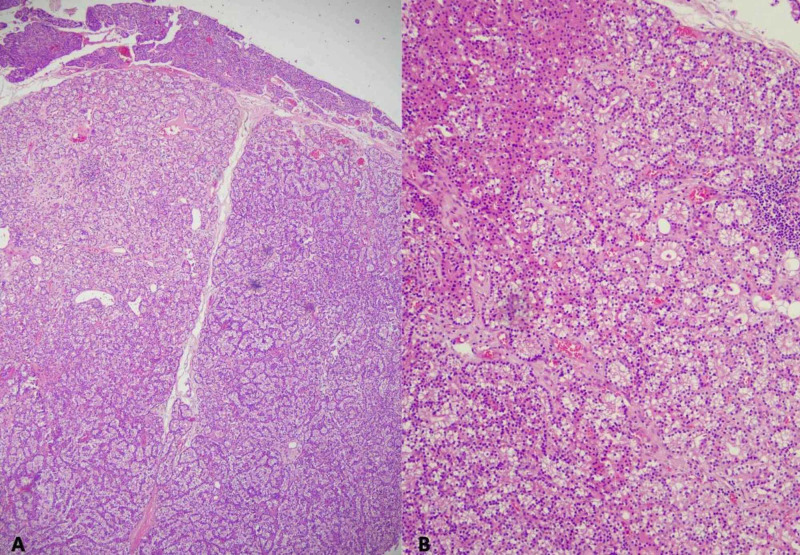
(A) Section shows a tumor with well-circumscribed borders. It is hypercellular and devoid of stromal adipose cells. A thin rim of normal residual parathyroid tissue is seen in the upper field (H&E, x40). (B) The tumor shows a follicular pattern, composed predominantly of chief cells with focal collection of oxyphil cells in the left upper field (H&E, x100).

The specimen was subjected to histopathological examination, which showed a tumor with follicular pattern, composed predominantly of chief cells with focal collection of oxyphil cells, confirming parathyroid adenoma (Figure 5).

Postoperatively, the patient was given intravenous antibiotics and analgesics for a period of seven days, and there was no untoward event noted. Levels of PTH and calcium decreased to normal range. Subsequently, the patient was referred back to department of urology for the management of renal calculi, where the patient underwent left anatrophic nephrolithotomy with left “double J” (DJ) stent placement. The serum calcium and PTH levels are being monitored every three months, and there is no recurrence in one-year follow-up.

## Discussion

Hypercalcemia can occur due to a multitude of clinical conditions; however, the differential diagnoses of hypercalcemia with increased PTH are limited. Conditions that may mimic PHPT include familial hypocalciuric hypercalcemia (rare), malabsorption syndromes, and certain medication use, such as thiazide diuretics, lithium, bisphosphonates, or denosumab therapy [2,5]. Although the majority of patients with PPD have sporadic disease, the workup should rule out multiple endocrine neoplasia (MEN) syndrome. It is important to rule out other causes for a high PTH due to low vitamin D and renal insufficiency (creatinine clearance <60 ml/min) and rarely Paget’s disease.

Elevated serum calcium levels in patients with PHPT may cause non-specific symptoms, such as anorexia, constipation, nausea and vomiting, depression, memory loss, fatigue, bone or muscle pains, and renal stones. Severe hypercalcemia may be life threatening as well leading to cardiac arrhythmias, coma, and death.

Renal stone is a major component of symptomatic PHPT. Globally, the occurrence of clinically overt stone disease has declined from 80% to 7%-20% due to the changing clinical picture of PHPT towards more milder or asymptomatic presentation [2]. However, when systematically evaluated the diagnostic prevalence of stone disease may be higher (25%-55%) [6]. Patients with PHPT with renal stones are often young males [2]. Hypercalciuria is an important contributor of renal stones [7]. Distinguishing nephrolithiasis from nephrocalcinosis in PHPT is often difficult, and prevalence of these two entities cannot be estimated separately due to lack of available literature [7]. In PHPT, the renal stones are predominantly composed of calcium phosphate, although calcium oxalate and mixed calcium stones are also common; however, uric acid stones are less often encountered [2].

Development of renal insufficiency in PHPT is also related to the severity and duration of hypercalcemia. In a cross-sectional study, 17% of 294 PHPT patients had estimated glomerular filtration rate (eGFR) below 60 ml/min [8]. The relative risks of developing renal stones and renal failure were 4.6% and 19.3%, respectively, in a cohort of mild asymptomatic PHPT patients [9]. 

Parathyroid adenomas, the most common cause of PHPT, are not usually palpable clinically, unless cystic, and rarely cause compressive symptoms. Diagnosis is based on biochemical and radiological tests, and confirmed by histopathology following surgery. Imaging modalities, such as ultrasound and 99mTc-sestamibi scan, are useful in visualization of diseased parathyroid [10]. Tc-99m sestamibi SPECT scintigraphy is an excellent modality as it is a single radiotracer based, non-invasive three-dimensional assessment of parathyroid adenoma with dual-phase acquisition capability. Sestamibi diffuses passively through cell membranes and accumulates almost exclusively in mitochondria. High mitochondria content in oxyphilic cells is considered to be the reason for the excessive tissue uptake in PHPT [11]. The role of sestamibi scan in diagnosis is limited by lack of nuclear medicine department at most hospitals in India, the use of radioisotopes, slow scan time, and poor anatomical localization if two glands lie in close proximity.

Sonography, on the other hand, is a cheap and widely available modality. It showed a characteristic discrete homogeneous hypoechoic mass posterior to the thyroid gland, even when the adenoma is as small as 1 cm [12]. A meta-analysis of 35 ultrasound and 14 sestamibi single photon emission computed tomography (SPECT) studies documented a pooled sensitivity of 76.1% and 78.9%, respectively. Independently, parathyroid ultrasonography has a sensitivity of 76%-87%, a positive predictive value of 93%-97%, and a diagnostic accuracy of 88% [13]. Additionally, ultrasound can identify concurrent thyroid nodules that may require biopsy prior to parathyroidectomy. Axial contrast-enhanced CT through neck helps localize parathyroid adenomas in 46%-87% cases, allowing accurate imaging for focused surgical techniques [14].

Surgical excision of parathyroid is considered both curative and standard treatment for PHPT, with first-time cure rates exceeding 95% [15]. In symptomatic patients or those with end-organ disease, such as low bone mineral density (BMD) or kidney stones, cohort studies have demonstrated that after successful parathyroidectomy, BMD improves substantially and the incidence of kidney stones declines; cognitive function may improve and cardiovascular disease rates and premature death also appear to decrease [16]. For these reasons, if no contraindications exists, all patients with symptomatic PHPT or in whom there is evidence of end-organ disease should receive parathyroidectomy. Bisphosphonates and hormone replacement therapy (HRT) are treatment options for those with PHPT where primary goal is skeletal protection, while the calcimimetic cinacalcet effectively lowers serum calcium and PTH levels in cases that are unsuitable for surgery; however, there is dearth of data demonstrating positive end-organ outcomes [17]. With the advent of the preoperative diagnostic techniques, concept of MIP has developed, and is considered better in terms of postoperative pain, scarring, early recovery, and decreased risk of postoperative hypoparathyroidism than traditional bilateral neck exploration [18]. 

## Conclusions

Hyperparathyroidism ought to be suspected and evaluated in all patients presenting with recurrent and bilateral renal calculi. A routine biochemical profile including serum calcium is useful in early screening for the condition. At centers where the radioisotope scanning is not available, preoperative diagnostic localization of parathyroid adenoma by ultrasonography is reliable and sufficient. Parathyroidectomy by minimally invasive technique is the standard of care and has high success rates in ameliorating the renal and skeletal manifestations of hyperparathyroidism.
